# Effects of Protein Corona on IAPP Amyloid Aggregation, Fibril Remodelling, and Cytotoxicity

**DOI:** 10.1038/s41598-017-02597-0

**Published:** 2017-05-26

**Authors:** Emily H. Pilkington, Yanting Xing, Bo Wang, Aleksandr Kakinen, Miaoyi Wang, Thomas P. Davis, Feng Ding, Pu Chun Ke

**Affiliations:** 1 ARC Centre of Excellence in Convergent Bio-Nano Science and Technology, Monash Institute of Pharmaceutical Sciences, Monash University, 381 Royal Parade, Parkville, VIC 3052 Australia; 2 Department of Physics and Astronomy, Clemson University, Clemson, SC 29634 USA; 3 Department of Chemistry, Warwick University, Gibbet Hill Coventry, CV4 7AL United Kingdom

## Abstract

Aggregation of islet amyloid polypeptide (IAPP), a peptide hormone co-synthesized and co-stored with insulin in pancreatic cells and also co-secreted to the circulation, is associated with beta-cell death in type-2 diabetes (T2D). In T2D patients IAPP is found aggregating in the extracellular space of the islets of Langerhans. Although the physiological environments of these intra- and extra-cellular compartments and vascular systems significantly differ, the presence of proteins is ubiquitous but the effects of protein binding on IAPP aggregation are largely unknown. Here we examined the binding of freshly-dissolved IAPP as well as pre-formed fibrils with two homologous proteins, namely cationic lysozyme (Lys) and anionic alpha-lactalbumin (aLac), both of which can be found in the circulation. Biophysical characterizations and a cell viability assay revealed distinct effects of Lys and aLac on IAPP amyloid aggregation, fibril remodelling and cytotoxicity, pointing to the role of protein “corona” in conferring the biological impact of amyloidogenic peptides. Systematic molecular dynamics simulations further provided molecular and structural details for the observed differential effects of proteins on IAPP amyloidosis. This study facilitates our understanding of the fate and transformation of IAPP *in vivo*, which are expected to have consequential bearings on IAPP glycemic control and T2D pathology.

## Introduction

Human islet amyloid polypeptide (IAPP) is a 37-residue peptide hormone that, along with insulin, plays an essential role in glycemic control^1^. IAPP is stored in soluble forms at millimolar concentrations in the islets of Langerhans before being released to the bloodstream. Accumulating evidence suggests that amyloid aggregation of IAPP is related to pancreatic β-cell death in type-2 diabetes (T2D), a debilitating disease impairing 368 million people worldwide^2^. *In vitro* studies have revealed that IAPP at micromolar concentrations can readily fibrillate into amyloids within hours, indicating that pancreatic β-cells endogenously inhibit IAPP aggregation^3^.

Earlier studies ascribed IAPP amyloids as the toxic species^4, 5^, while more recent studies pointed to IAPP oligomers as the causative toxic agent for β-cell loss^6^. The ambiguities surrounding IAPP toxicity largely stem from the difficulty in isolating IAPP monomers from oligomers, protofibrils and amyloids due to the rapid fibrillation kinetics of the peptide, as well as the complex intra- and extra-cellular environments of the IAPP species where peptides, proteins, lipids and fatty acids occur in abundance^6^. In consideration of the changing conformation and hydrophobicity of IAPP during its fibrillation process, it is reasonable to postulate that the molecular ligands encountered by IAPP from cradle to grave may exert effects on the physical and biological identities of the IAPP species.

Surprisingly, little is known concerning IAPP-protein interactions and their biological and pathological implications. Several co-factors, including serum amyloid P (SAP) component^7^, apolipoprotein E (ApoE)^8^, and glycosaminoglycans (GAGs), in particular heparan sulfate proteoglycans^9^, have been shown to associate with IAPP amyloid deposits *in vivo*. GAGs are also known to enhance fibrillation of IAPP^10, 11^, and can promote aggregation of incompletely processed IAPP^12^. Secretory chaperones and serum albumin have also demonstrated some capacity in preventing IAPP fibrillation *in vitro* ^11, 13^. However, protein association with IAPP does not necessarily affect residual IAPP-mediated cytotoxicity; in the cases of ApoE and GAGs, only the latter is implicated in IAPP toxicity^14, 15^.

In this study we examined the binding of both fresh IAPP and mature IAPP amyloids with two model proteins, lysozyme (Lys) and alpha lactalbumin (aLac). Lys and aLac are homologous proteins with similar tertiary structures (14 kDa, 41% helical and 9% beta sheets) while carrying opposite net charges. Lys is an enzyme commonly found in saliva and tears responsible for hydrolyzing peptidoglycans in bacterial cell wall while aLac from mammal milk regulates lactose biosynthesis. Both Lys^16^ and aLac^17^ can also be found in circulation. We used thioflavin T (ThT), circular dichroism (CD), and high-resolution transmission electron microscopy (TEM) to assess IAPP aggregation and fibril remodeling, and performed a viability assay with human umbilical vein endothelial cells (HUVECs) to evaluate the toxicities of fresh IAPP and IAPP amyloids in the presence of the model proteins. Specifically, co-incubated with IAPP peptides at a 1:1 molar ratio Lys inhibited IAPP aggregation with no visible fibril formation while aLac induced amorphous aggregation containing significant beta-sheet contents. TEM imaging revealed that both aLac and Lys bound mature fibrils and binding of aLac led to fibril softening. Surprisingly, the cell viability study indicated that Lys enhanced but aLac reduced the cytotoxicity of IAPP peptides, whereas binding of either Lys or aLac with mature IAPP fibrils mitigated the fibril toxicity.

To gain a molecular insight into the binding of homologous Lys and aLac proteins with both soluble IAPPs and insoluble amyloid fibrils and to understand the corresponding differential effects on IAPP-mediate cytotoxicity, we performed discrete molecular dynamics (DMD) simulations to examine the structure and dynamics of a selected few of relevant molecular systems. DMD has been successfully applied in our previous studies of IAPP aggregation inhibition by polyphenol^18^, graphene oxide^19^, and dendritic polymers^20^, and offered molecular details in this study especially concerning inhibition of IAPP fibril formation and elevation of IAPP toxicity in the presence of Lys. Both Lys and aLac were found to bind positively charged IAPP monomers *in silico*. Simulations of multiple proteins and peptides at a 1:1 molar ratio showed that one Lys protein could bind multiple IAPP peptides and the increased net charge of the IAPP-Lys clusters prevented further peptide association. Therefore, Lys binding stabilized IAPP oligomers, known to be more toxic than the fibrils, and resulted in increased toxicity of IAPP. In contrast, aLac co-aggregated with IAPPs by forming large protein-peptide complexes through electrostatic attraction, yielding the experimentally observed amorphous aggregates with less toxicity compared to IAPP alone. Our simulations also showed that both Lys and aLac could bind the fibrils, which likely reduced cell exposure to the fibrils and hence mitigated cytotoxicity. To account for the higher protein to IAPP ratio in physiological conditions, we performed additional DMD simulations with an 8:1 protein to IAPP molar ratio. Crowded with proteins (either aLac, Lys, or their mixture), the IAPP peptides tended to bind one protein, which minimized the formation of both toxic oligomers and amorphous aggregates. Together, our combined *in silico* and *in vitro* study has revealed the contrasting effects of proteins on IAPP amyloid aggregation, fibril remodeling and cytotoxicity depending on the physicochemical properties as well as the relative concentrations between the proteins and IAPP peptides, pointing to a natural defense mechanism of biological systems in mitigating the toxicities elicited by amyloidogenic species.

## Results and Discussion

### Biophysical characterizations of protein binding on IAPP aggregation and fibril remodelling

#### Zeta potentials

IAPP (IEP: 8.9) assumed a zeta potential of +36.8 mV at neutral pH, while Lys (IEP: 9.1) and aLac (IEP: 4.2) displayed zeta potentials of +12.1 mV and −14.6 mV, respectively. Based on their primary sequences, the corresponding net charges of monomeric IAPP, Lys and aLac are +2e, +7e and −7e. The observed higher zeta potential of IAPP with a smaller net charge was possibly due to their rapid formation of oligomers and protofibrils in solutions.

#### ThT and CD assay quantifications of IAPP fibrillation inhibition by proteins

**Figure 1:**
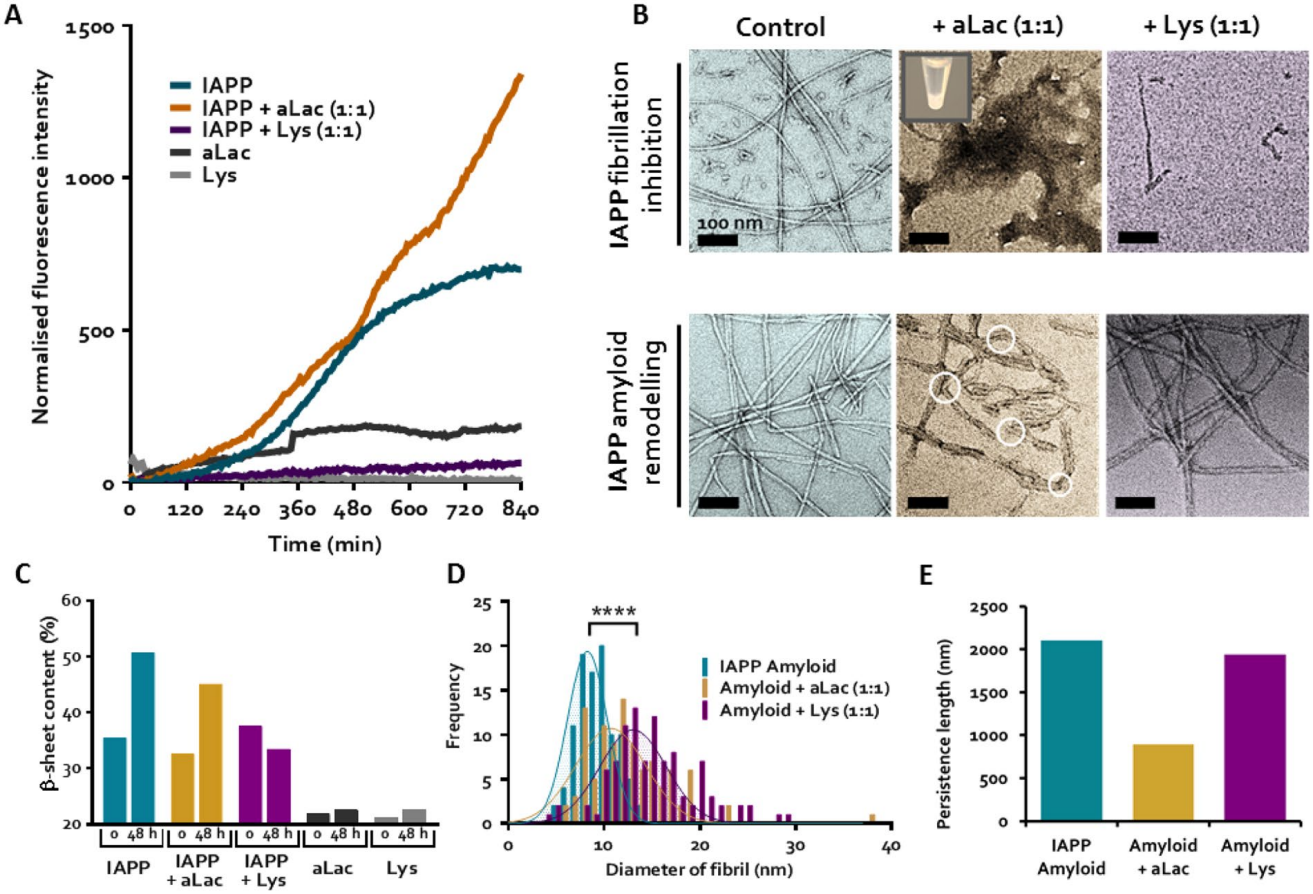
IAPP fibrillation inhibition and amyloid remodelling by lysozyme (Lys) and α-lactalbumin (aLac). (**A**) Thioflavin T (ThT) fluorescence assay shows IAPP fibrillation in the presence of Lys and aLac over 14 h. (**B**) TEM images of IAPP fibrillation inhibition (upper panel) and IAPP amyloid remodelling (lower panel) mediated by Lys and aLac at a 1:1 molar ratio after incubation in Milli-Q water for 24 h at 25 °C. Formation of a visible IAPP-aLac precipitate (upper middle panel, inset) and soft aLac-amyloids (lower middle panel, white circles) are shown. Scale bars: 200 nm. (**C**) Circular dichroism (CD) shows the β-sheet content of IAPP in the presence of aLac and Lys, in addition to the aLac and Lys controls, over 48 h. (**D,E**) Analysis of IAPP amyloid fibril diameter (**D**) and persistence length (**E**) in the presence or absence of Lys and aLac. IAPP concentration = 25 μM for all experiments. ****p < 0.0001 *(unpaired t-test, n* = *100)*.

IAPP, aLac and Lys were incubated with the amyloid-sensitive ThT dye to quantify the rate and kinetics of IAPP fibrillation over 14 h (Fig. 1A), in addition to visualising change in protein secondary structure up to 48 h through CD spectroscopy (Fig. 1C). It was demonstrated that IAPP alone remained in the energetically unfavourable nucleation, or lag, phase up to 5 h, before entering the elongation phase, and by 14 h was within the saturation phase (Fig. 1A). This in turn was complemented by an increase of IAPP β-sheet content from 35.4% at 0 h to 50.7% after 48 h (Fig. 1C), indicative of the increasing prevalence of β-sheet rich amyloid species.

The secondary structure of IAPP mixed with aLac showed an analogous increase in β-sheets to the IAPP control after 48 h (Fig. 1C), which was not complemented in aLac (Fig. S1). Comparative to IAPP alone, however, IAPP-aLac did not show evidence of sigmoidal fibrillation, and the increase in ThT fluorescence followed a more linear trend (Fig. 1A). IAPP-aLac also attained the largest increase in ThT fluorescence overall, indicating a promotional effect of aLac on IAPP aggregation. The non-sigmoidal fibrillation trend, however, was consistent in all experiments; the combined intensity and kinetics observations therefore suggest formation of unstructured, amorphous IAPP aggregates in the presence of aLac. Conversely, complete inhibition of IAPP was observed in the presence of Lys over the 14 h sampling period for ThT (Fig. 1A); concordantly, no increase in the percentage of IAPP β-sheet structure in IAPP:Lys was seen after 48 h (Fig. 1C). Each protein therefore mediated opposing effects on IAPP fibrillation, both in terms of β-sheet content and kinetic behaviour.

#### High-resolution TEM imaging of IAPP fibrillation inhibition and remodelling by proteins

TEM imaging was perfromed to complement ThT analysis through visualization of the aggregation products (Fig. 1B, upper panel), and also to examine IAPP amyloid remodelling mediated by aLac or Lys (Fig. 1B, lower panel). The TEM images were further analyzed using statistical software FiberApp^21^ to determine the morphological and mesoscopic changes of mature IAPP amyloid fibrils incubated with aLac or Lys, including fibril diameter (Fig. 1D) and persistence length (fibril stiffness) (Fig. 1E).

By 24 h incubation of IAPP alone, both long fibrils and smaller aggregates (also known as protofibrils) appeared (Fig. 1B, upper panel). Neither of these structures was observed in the presence of aLac or Lys, however. Specifically, IAPP fibrillation was completely inhibited by Lys, with only a few small, worm-like structures present after 24 h. Electrostatic repulsion between the positively charged IAPP and Lys likely compromised IAPP-IAPP interactions. In comparison, the IAPP-aLac mixture was mainly present as large amorphous aggregates after 24 h, which was also observed as off-white precipitates in solution (Fig. 1B upper panel, inset). Based on the ThT assay (Fig. 1A), the amorphous aggregates contained significant beta-sheet structures. Considering the opposing charges of IAPP and aLac, it is likely that electrostatic attraction with aLac strongly perturbed IAPP self-assembly into fibrils, while favoured formation of amorphous structures.

Alternatively, formation of non-fibrillar species has been observed in both IAPP and Aβ amyloidogenesis when monomers interact with hydrophobic surfaces^22, 23^. This suggests that IAPP-aLac could act as a seed, promoting contact between monomeric or low-order oligomeric IAPP species, yet directing aggregation off-pathway.

Mature IAPP amyloids formed by IAPP alone after 30–60 days of incubation were long and semi-flexible (Fig. 1B, lower panel). The presence of some shorter species is attributed to the cross-linking and subsequent gelation of mature IAPP amyloids^24^, which can result in some fibril breakage during pipetting from the stock. Statistical analysis of these fibrils revealed an average diameter of ~8.9 nm (Fig. 1D) and an average persistence length of ~2,100 nm (Fig. 1E). When incubated with aLac and Lys, IAPP amyloids underwent remodelling, as indicated by changes in fibril diameter and morphology compared to IAPP amyloids alone (Fig. 1B, lower panels). Both aLac and Lys interacted with the fibrils, mediating a significant shift in average fibril diameter (Fig. 1D) from approximately 8.9 nm (IAPP amyloid control) to 12.1 nm (aLac) and 14.5 nm (Lys), correspondingly. Interestingly, interaction with aLac halved the persistence length of amyloid fibrils (Fig. 1E) indicating significant fibril softening, while no difference was seen in IAPP stiffness with Lys binding.

The endogenous inhibition of Aβ(1–40) fibrillation mediated by several non-chaperone proteins, including aLac and Lys, has been previously reported^25^. It was shown that fibrillation of the negatively charged Aβ was inhibited by aLac and Lys at a ~1:1 molar ratio, yet both proteins were unable to promote disaggregation of pre-formed Aβ fibrils. In the case of IAPP, although both aLac and Lys inhibited the fibrillation, aLac promoted the formation of large amorphous aggregates with significant beta-sheet content while Lys prevented any formation of aggregates that can be detected by either ThT and TEM imaging. The differential impact on IAPP amyloid aggregation and remodelling of IAPP amyloids by aLac and Lys in our study suggests that both electrostatic and hydrophobic interactions between proteins and IAPP can alter the intrinsic properties of IAPP amyloids; as such, it is likely that IAPP amyloids can facilitate interactions with a multitude of components in the environmental milieu. Formation of a ‘protein corona’^26, 27^ on IAPP amyloids *in vivo* is likely to have a significant effect on IAPP amyloid toxicity, and merits further investigation.

### Effects of protein binding on the viabilities of fresh IAPP and IAPP amyloids

**Figure 2:**
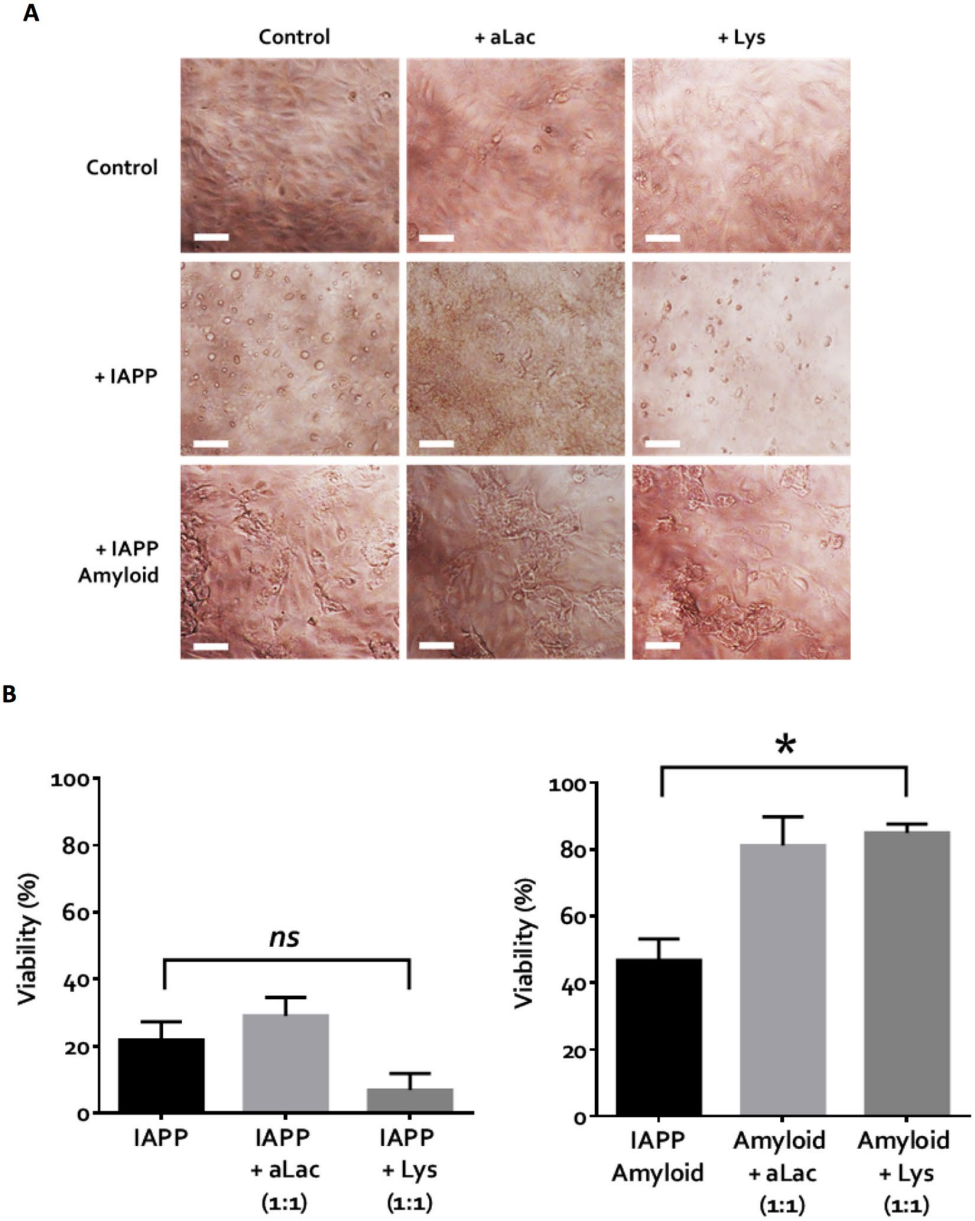
Viability of HUVECs exposed to 25 μM IAPP and amyloids in the presence of α-lactalbumin (aLac) and lysozyme (Lys) after 24 h. (**A**) Bright-field images reveal extensive cell death in the presence of IAPP, and some loss of cells with IAPP amyloids. (**B**) The calcein-AM viability assay demonstrates high toxicity of IAPP (left) and, to a lesser extent, IAPP amyloids (right). Significant mitigation of toxicity in amyloids was observed when pre-treated with aLac and Lys. *Scale* = 25 μm; *p < *0.05 (one-way ANOVA, n* = *3)*.

Bright-field imaging (Fig. 2A) showed healthy control HUVECs as highly confluent and endothelial-like in morphology. Microscopic examination was additionally complemented by the calcein-AM assay (Fig. 2B) for quantitative measurement of cell viability against an untreated control. Large-scale cell damage and death was observed with IAPP, regardless of the presence or absence of aLac or Lys (Fig. 2A, middle row). Viability data (Fig. 2B, left panel) for IAPP (22% viability), IAPP-aLac (29%) and IAPP-Lys (7%) further demonstrated that aLac and Lys, though capable of inhibiting IAPP fibrillation, had no mitigating effect on the peptide toxicity. In fact, IAPP-Lys mixture showed notably higher toxicity in HUVECs than IAPP alone, suggesting that Lys binding might stabilize IAPP as a highly toxic low molecular weight species (Fig. 1). The reduced toxicity of IAPP-aLac mixture was likely due to the fact that the formed amorphous aggregates were very large and precipitated (Fig. 1B, IAPP-aLac inset), reducing their exposure and subsequent toxic effects to cells.

Interestingly, different phenomena were observed when aLac and Lys were incubated with IAPP amyloids. Large deposits of mature amyloid aggregates were seen with each condition (Fig. 2A, lower row); however, extensive cell destruction, as visualized with IAPP, was not present. In amyloid-aLac and amyloid-Lys, healthy cells were observed. This effect is corroborated by the viability data (Fig. 2B, right panel); the relatively lower level of toxicity mediated by IAPP amyloids (47% viability) was significantly reduced in the presence of both aLac (81% viability) and Lys (85% viability). Therefore, our results suggest that the formation of aLac or Lys ‘corona’ on the amyloid fibril surface likely screened the amyloid-cell interactions and thus reduced the amyloid fibril-induced cytotoxicity.

In a previous study, we explored the complex nature of IAPP fibrillation species and their associated toxicities^28^. We demonstrated that fibrillating, stabilized oligomeric and mature amyloid IAPP species were toxic to different extents to pancreatic beta cells *in vitro*. This study supports this narrative with a different cell line and hence a different *in vitro* IAPP environment, and additionally provides new insight. To better understand the differential effects of aLac and Lys binding on IAPP aggregation, fibril remodelling and toxicity, we next performed atomistic DMD simulations to study the binding of aLac or Lys with both soluble IAPP monomers and amyloid fibrils.

### DMD simulations of protein binding with soluble IAPP and IAPP fibrils

**Figure 3:**
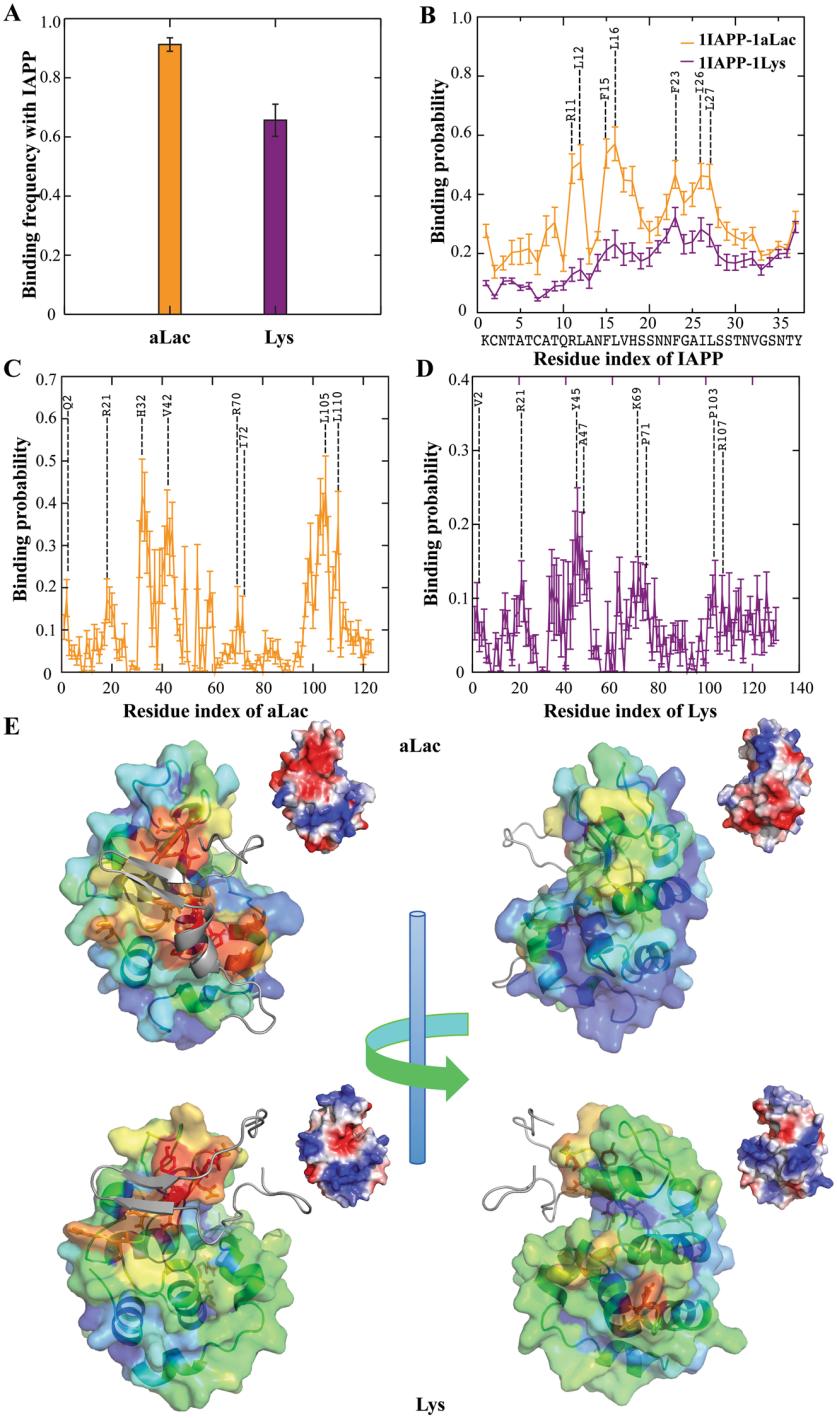
Binding of an IAPP monomer with aLac and Lys. (**A**) Binding frequencies of IAPP monomers with either aLac or Lys during the course of simulations. (**B**) Binding probability of each IAPP residue with either aLac or Lys was derived from the IAPP-protein complexes in DMD simulations. Similarly, the binding probabilities of residues in aLac (**C**) and Lys (**D**) with IAPP were also computed. The residues with peaks values were highlighted, where the residue indices were re-numbered from 1 in each corresponding sequence. (**E**) Typical snapshot structures of IAPP-protein complexes. The molecular surfaces of both aLac and Lys were shown to highlight their binding with IAPP (colored grey in cartoon representation), where each protein residue was colored from blue (low) to red (high) according to its binding probability to IAPP as in panels C &D. Two different views were given to illustrate multiple IAPP-binding sites on the protein surfaces, and corresponding electrostatic potential surfaces (estimated with PyMol) were shown in the inset.

We first performed DMD simulations of IAPP monomers binding with either aLac or Lys. For each molecular system here and later in the study, 20 independent simulations were performed at 300 K with different initial inter-molecular distances and orientations (Methods). Each simulation lasted 50 ns so that an accumulative 1 μs of total DMD simulations was done for each molecular system. Despite the intrinsically high conformational flexibility of IAPP, structural analysis of the peptide along simulation trajectories (e.g., radius of gyration and secondary structure contents in Fig. S2) suggested that an equilibrium or “steady-state” in terms of conformational dynamics was achieved in simulation. We found that IAPP could bind to both proteins, with aLac having a higher binding frequency to IAPP than Lys (Fig. 3A). The stronger binding of aLac with IAPP is mainly due to their opposite net charges. Based on the bound complex structures, we computed the binding probability of each IAPP residue with the proteins (Fig. 3B). The peptide residues had an overall higher binding probability with aLac than with Lys. As highlighted in Fig. 3B, the residues with peak binding values along the peptide sequence were mostly hydrophobic, with the positively charged R11 also displaying strong binding to aLac. Similarly, we computed the binding probability of the protein residues with IAPP (aLac in Fig. 3C and Lys in Fig. 3D). Since Lys and aLac were structural homologs with ~35% sequence identical (Smith-Waterman algorithm^29^), the residue-wise IAPP binding profiles for aLac and Lys shared some similarities (e.g., residues around N-terminal, 20, and 30–50 in Fig. 3C,D). One major difference was the high peak around residues 100–110 for aLac that was absent for Lys. The residues of aLac with high IAPP-binding values were mostly hydrophobic (Fig. 3C), while the IAPP-binding residues in Lys were more hydrophilic (Fig. 3D). Typical snapshot structures, where the protein residues were color-coded according to their IAPP-binding frequencies (Fig. 3E), indicated the similarities and differences of peptide binding with aLac and Lys. The C-terminal of IAPP tended to bind to a clef of aLac and Lys (e.g., residues 30–50), while the N-terminal of IAPP made contact with aLac (i.e., residues 100–110 as in Fig. 3C) but not with Lys. A different viewpoint of the complex structures shows other IAPP-binding sites, suggesting a relatively non-specific binding of IAPP with either aLac or Lys. Therefore, our simulations revealed that IAPP-aLac binding was dominated by hydrophobic interactions while IAPP-Lys binding was mainly driven by polar interactions. We note that the relative contributions of various IAPP-protein interactions derived from simulations can be validated experimentally by site-directed mutagenesis of corresponding residues and subsequent measurements of peptide binding. As our current study is focused on the effects of protein binding on IAPP aggregation, these protein-peptide binding interactions warrant future investigations.

**Figure 4:**
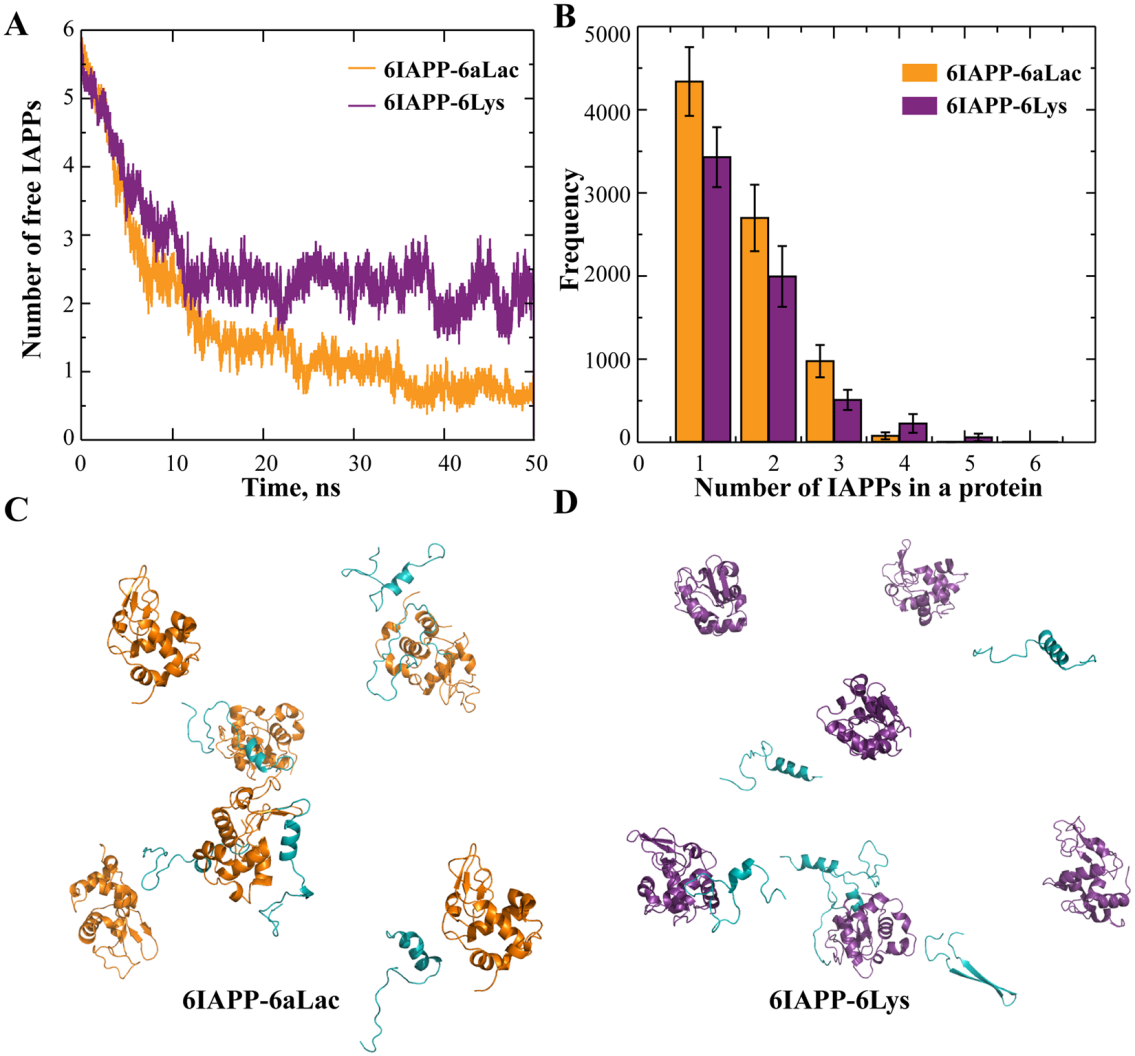
Binding of multiple IAPPs with multiple proteins of fixed positions. (**A**) Number of unbound IAPP peptides averaged over independent simulations as a function of simulation time. (**B**) Histograms of the number of peptides bound to either aLac or Lys were computed from the last 12.5 ns of corresponding simulations. (**C**,**D**) Snapshot structures of IAPP (cyan) peptides binding with both aLac (orange; **C**) and Lys (purple; **D**), where both peptides and proteins were in cartoon representation.

To elucidate the binding of multiple IAPP peptides with multiple proteins, we tested molecular systems with 6 proteins and 6 peptides. Considering the significant computational cost to simulate such large systems, we allowed peptides free to move while fixed the coordinates of all proteins. We found that IAPPs rapidly bound to either aLac or Lys, with only small fractions of unbound IAPP at the end of the simulations (Fig. 4A). To understand how the peptides were bound to proteins, we performed a cluster formation analysis of snapshot structures along the simulation trajectories: two molecules formed a cluster if they were in contact (i.e., making at least one inter-molecular contact) and two molecules belonged to a cluster if both of them were in contact with another molecule. For simulations of each protein, we used the last 12.5 ns of 20 independent simulations where the binding was apparently equilibrated (Fig. 4A), and computed histograms of the number of IAPP peptides forming a cluster with a single protein (Fig. 4B). While the probability of finding a single IAPP bound a single protein was the highest for both aLac and Lys, multiple IAPPs could bind to a single protein with high probabilities (e.g., snapshot structures in Fig. 4C,D). Therefore, both aLac and Lys could bind IAPP oligomers.

**Figure 5:**
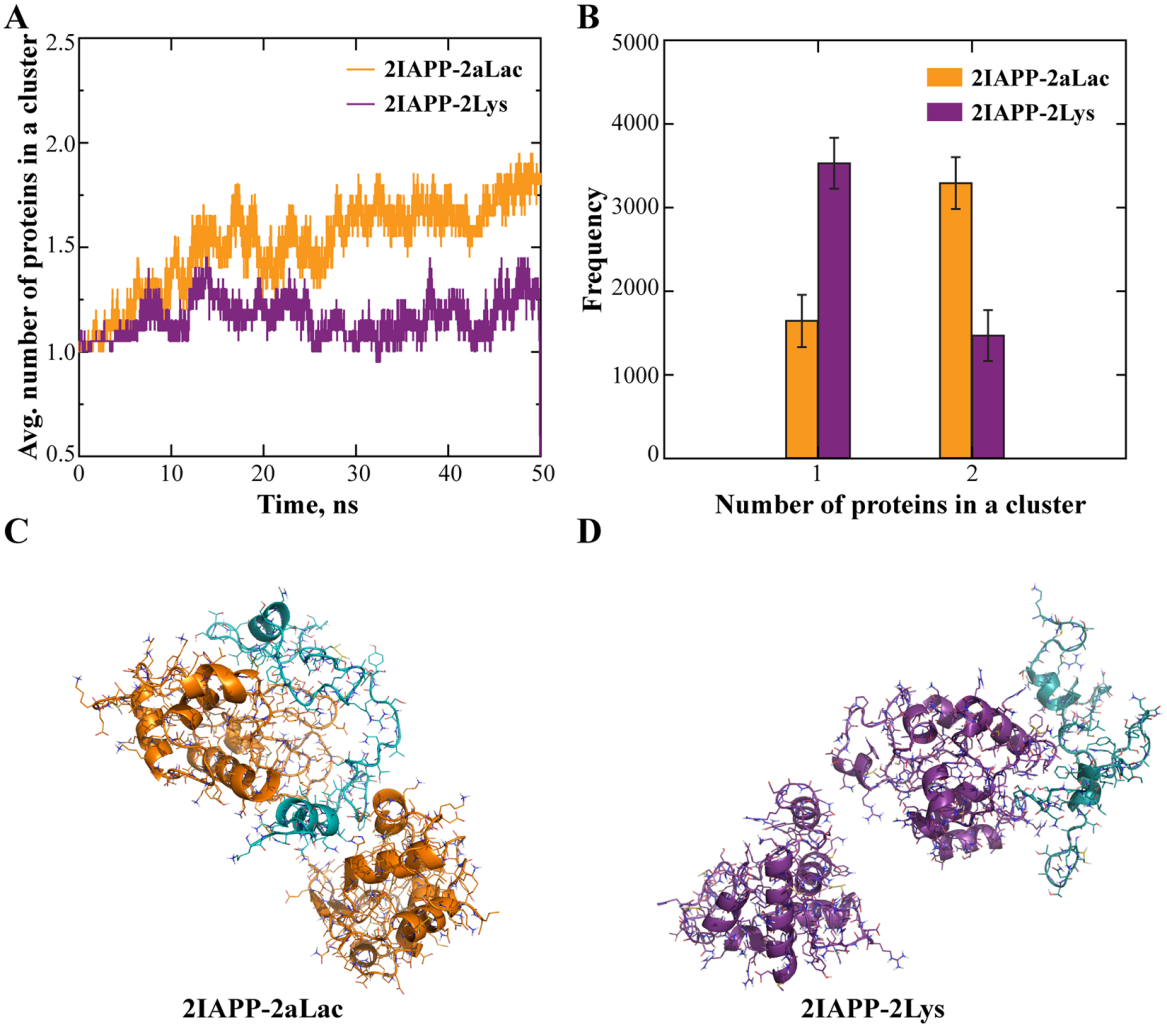
Binding of two IAPPs with two free proteins. (**A**) Average number of proteins belonging to a protein-containing cluster. (**B**) Histogram of the number of proteins in a protein-containing cluster. (**C,D**) Snapshot structures of IAPP (cyan) peptides binding with aLac (orange; **C**) and Lys (purple; **D**), taken from DMD simulations.

Since the protein coordinates were fixed in the above simulations, we were not able to observe potential co-aggregation of both proteins and peptides. Next, we allowed proteins to move in DMD simulations, but used smaller molecular systems for them to be computationally tractable. We included 2 proteins and 2 peptides in the simulations (Fig. 5), and monitored the number of proteins forming a cluster to evaluate the corresponding co-aggregation with the peptides. Averaging over 20 independent simulations, we computed the average number of proteins in a protein-containing cluster, *N*_*p*_, as a function of simulation time (Fig. 5A). Given only two proteins in these simulations, the value of 1 corresponded to the two proteins separated and 2 for the case of protein aggregation. Starting from separated proteins, *Np* of the aLac-IAPP mixture increased to nearly 2 while *N*_*p*_ of the Lys-IAPP mixture fluctuated near 1. Two aLac tended to form one cluster but Lys stayed more separated (Fig. 5B), as illustrated by snapshot structures from the corresponding simulations (Fig. 5C,D). We also performed similar simulations and analyses of 2 proteins interacting with 4 IAPP peptides and obtained similar results (Fig. S3); the tendencies of aLac co-aggregating with IAPP and Lys staying separated were stronger, consistent with experimental observations. Specifically, aLac co-aggregated with IAPP to form large amorphous aggregates while Lys binding stabilized the small molecular weight oligomers that were highly toxic to HUVECs (Figs 1 and 2).

**Figure 6:**
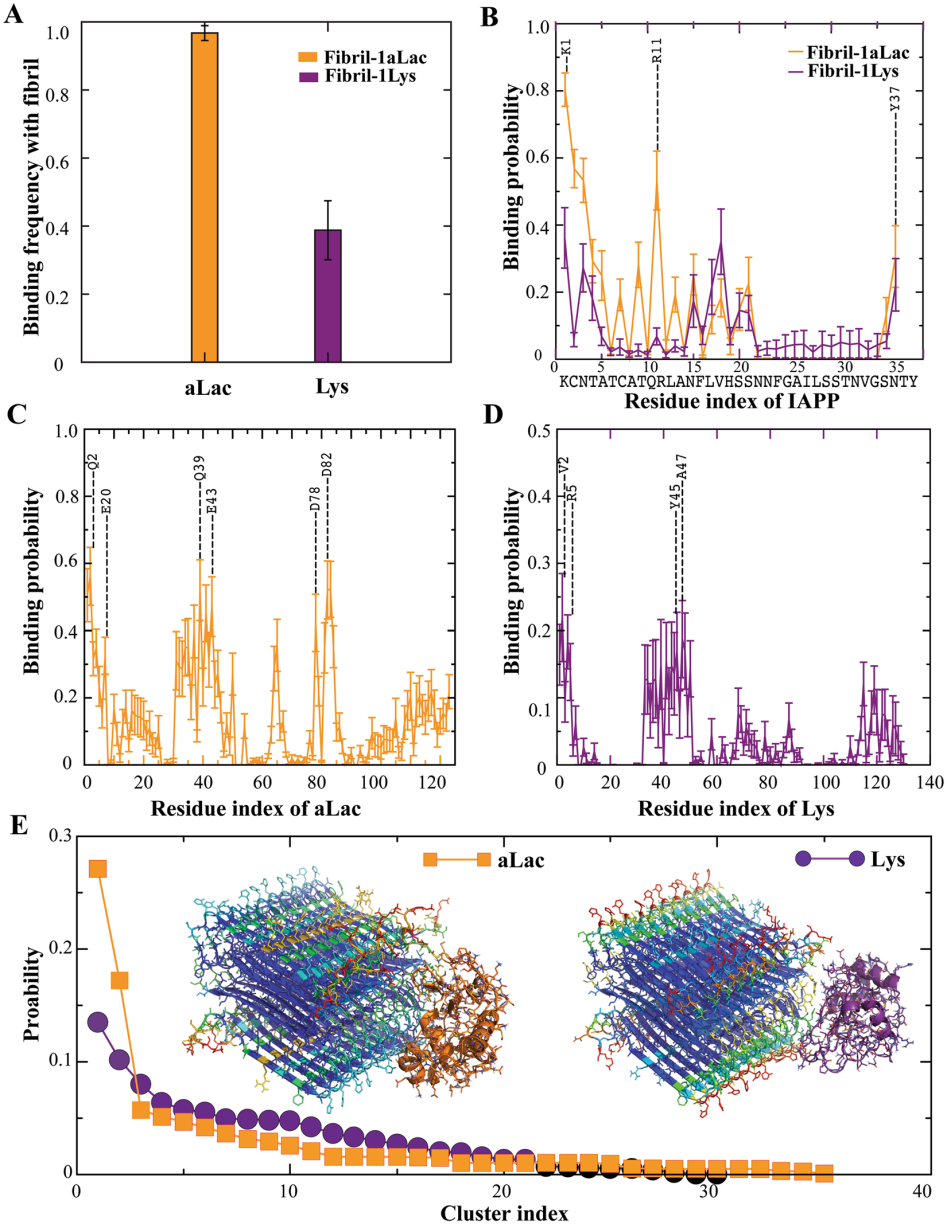
Binding of proteins with IAPP amyloid fibril. (**A**) Binding frequencies of either aLac or Lys with an IAPP fibril during the course of simulations. (**B**) Binding probability of each IAPP residue in the fibril with either aLac or Lys was derived from the IAPP-protein complexes. Similarly, the binding probabilities of residues in aLac (**C**) and Lys (**D**) with the IAPP fibril were also computed. The residues with peaks values were highlighted, where the residue indices were re-numbered from 1 in each corresponding sequence. (**E**) Normalized protein-fibril binding cluster sizes as a function of cluster index. The centroid structures of the top cluster for both aLac-fibril and Lys-fibril binding were shown in the inset. Both proteins and peptides were shown in cartoon representation with sticks and the IAPP residues in the fibril were colored according their corresponding binding frequencies to either aLac (orange) or Lys (purple).

We subsequently simulated binding of the two types of proteins with IAPP amyloid fibril. The two-layered beta-sheet fibril structure with each protofibril containing ten IAPP peptides were constructed based on solid-state NMR constraints (Methods)^30^. Considering the system’s complexity, only the unstructured loops and the sidechains of solvent exposed residues in the beta-sheets in addition to proteins were allowed to move in the simulations. We found that aLac elicited stronger binding to the fibril than Lys (Fig. 6A). The binding of proteins with IAPP fibrils (Fig. 6) was very different from their binding with soluble IAPP (Fig. 3). For example, aLac primarily bound to the charged IAPP residues in the fibril (e.g., K1 and R11 in Fig. 6B), which is expected since IAPP amyloid with multiple peptides forming in-registered beta-sheets was highly charged around these residues. Similarly, the aLac residues with high binding probability to the fibrils were mainly negatively charged residues (Fig. 6C). For the binding between Lys and the IAPP fibril, the residues with high binding probabilities in either the fibril (Fig. 6B) or Lys (Fig. 6D) included both polar and hydrophobic residues. Most of the C-terminal region of IAPP (except Y37) was buried at the protofibril interface of the fibril, thus avoiding interactions with the proteins (Fig. 6B).

To characterize the binding structures of either aLac or Lys with the IAPP fibril, we constructed the structural ensembles of protein-fibril complexes by evenly selecting snapshot structures from the simulations: 3,079 snapshot structures for Lys-fibril binding and 7,672 for aLac-fibril binding due to the stronger binding of aLac with the fibril (Fig. 6A). For each ensemble, we performed a clustering analysis with pair-wise center-of-mass distances of the proteins to group similar protein-fibril binding poses. With a cut-off distance of 1 nm, we obtained 30 clusters for Lys and 35 for aLac and computed the cluster size distributions normalized by the total number of structures in each ensemble (Fig. 6E). For aLac-fibril binding, the top two clusters contained ~45% of all snapshot structures while the rest of the clusters had small number of structures (the centroid structures of the top two clusters were shown in Fig. S4). In the case of Lys-fibril binding, the cluster sizes were more evenly distributed (the centroid structures of the top four clusters were shown in Fig. S5). Therefore, Lys tended to bind more evenly on the fibril surface while aLac had more specific binding sites on the fibril surface. This result explains the experimentally observed difference in fibril diameters remodelled by protein binding (Fig. 1D) – fibrils evenly covered with Lys resulted into larger diameters compared to fibrils bound by aLac at specific regions.

Because the fibrils were not allowed to move in our simulations, we could not capture the experimentally observed softening of fibrils upon aLac binding, which was likely due to the strong electrostatic-driven aLac-fibril binding (Fig. 6, Fig. S4). Given a high number of negatively charged residues in aLac (8 Asp and 12 Glu) and the geometrical incompatibility between the globular protein and the cylindrical fibril, such strong inter-molecular attractions may induce strain along the fibril, resulting in fibril bending and reduced fibril persistence length (Fig. 1E).

## Conclusion

We have applied ThT fluorescence assay, CD spectroscopy, TEM imaging, cell viability assay and atomistic DMD simulations to examine the effects of protein binding on IAPP amyloidosis, including IAPP aggregation kinetics and dynamics, fibril remodelling and cytotoxicity. We found that soluble IAPP could bind to both cationic Lys and anionic aLac, driven by either hydrophilic or hydrophobic interactions (Fig. 3). Since IAPP peptide is intrinsically disordered with high conformational flexibility, IAPP could bind many co-localizing proteins in general. Without imposing any prior assumptions into DMD simulations, protein binding with aLac and Lys displayed drastically different effects on IAPP self-association *in silico* due to their distinct physicochemical properties. Specifically, aLac and IAPP co-aggregated forming a large IAPP-aLac molecular complex (Fig. 1B), driven by strong binding affinity (Fig. 3) and opposite charges. The large aggregates formed by IAPP and aLac mixture elicited low toxicity to HUVECs. Lys, on the other hand, could bind and stabilize IAPP oligomers (Fig. 4B,D), where increased electrostatic repulsion prevented further aggregation of these clusters (Fig. 5 & Fig. S3). Stabilization of low molecular weight oligomers of IAPP by their associations with Lys resulted in increased toxicity of IAPP as toxic oligomers were transiently formed (Fig. 2). Interestingly, protein binding of IAPP fibril - i.e. formation of amyloid coronae - always reduced the relatively low toxicity of the fibril, suggesting a new mechanism for mitigating IAPP toxicity *in vivo*.

Given the over-abundance of globular proteins in the physiological medium (e.g., the blood circulation), we simulated 6 IAPPs interacting with 48 fixed proteins (Fig. S6). Compared to the simulations of 6 IAPP with 6 fixed proteins at a 1:1 molar ratio (Fig. 4), we found that majority of IAPPs bound one protein as in a monomeric form (Fig. S6B–E). Therefore, despite the general binding of IAPP with globular proteins and the potential of proteins in promoting toxic IAPP species at comparable protein/IAPP concentrations, a crowed environment with abundant globular proteins may inhibit the formation of IAPP oligomers. Further exploration is necessary to fully elucidate environmental proteins that may contribute to IAPP toxicity both in the pancreas and in circulation. Together, this new protein corona paradigm facilitates our understanding of the fate and transformation of IAPP *in vivo*, which may have consequential bearings on IAPP glycemic control and T2D pathology.

## Methods

### Materials

Human islet amyloid polypeptide (IAPP) (disulfide bridge: 2–7; MW: 3,906; 37-residue: KCNTATCATQRLANFLVHSSNNFGAILSSTNVGSNTY) was obtained as lyophilized powder from AnaSpec. Lysozyme (Lys) (from chicken egg white; MW: 14,300) and α-lactalbumin (aLac) (calcium depleted, from bovine milk; MW 14,178) were obtained from Sigma Aldrich. IAPP, aLac and Lys were weighed on a Cubis MSE balance (Sartorius, 0.01 mg resolution), dissolved in Milli-Q water (pH 6.5) to a concentration of 200 µM and used immediately for zeta potential, ThT, TEM and viability assay sample preparations. Pre-formed IAPP amyloids (30–60 days old in Milli-Q water, room temperature) were kept at a stock concentration of 200 μM. Thioflavin T (ThT) dye (Sigma) was dissolved in Milli-Q water to form a 250 μM stock solution immediately prior to use in ThT sample preparations. Calcein-AM dye (Sigma) was kept in a 1 mM stock solution in DMSO at −20 °C.

### Zeta potential

The zeta potentials of IAPP, Lys and aLac were determined using a dynamic light scattering device (Zetasizer Nano S90, Malvern Instruments) at room temperature. The concentration of IAPP was 25 μM while the concentrations of Lys and aLac were both 7.9 μM.

### Thioflavin T assay

IAPP, aLac and Lys were added to a black/clear bottom 96 well plate (Costar) to a final IAPP concentration of 25 μM, and final aLac and Lys concentrations of 25 μM, representing IAPP:protein ratios of 1:1. Thioflavin T (ThT) dye (Sigma Aldrich) was added to all wells to a concentration of 25 μM, and the remaining volume made up to 100 μL with Milli-Q water. The plate was kept at 25 °C and read on a Flexstation 3 plate reader (Molecular Devices) every 5 min for a total of 14 h (169 readings), with wells excited at 440 nm and the emission read at 485 nm.

### Circular dichroism spectroscopy

IAPP, aLac and Lys at equimolar concentrations (25 μM) were read after 0 and 48 h incubation at room temperature on a Chirascan CD spectrometer (Applied Photophysics) across a wavelength range of 190~260 nm. Final spectra were an average of three reads, which were then normalised against background signal and, for IAPP:protein samples, against individual protein controls at each respective time point. The percentage of β-sheet content in each protein was derived through deconvolution of normalised spectra using CDNN software.

### Transmission electron microscopy

IAPP, IAPP amyloids, aLac and Lys (25 μM; IAPP:proteins =1:1) were incubated in Milli-Q water at 25 °C for 24 h. A 10 μL aliquot was then taken and placed on 400 mesh carbon-coated formvar copper grids (ProSciTech) that were glow-discharged for 15 s to promote hydrophilicity. Sample adsorption was undertaken for 60 s, then drawn off on filter paper. Grids were washed twice in 10 μL Milli-Q water. 5 μL of 1% uranyl acetate (in water) was utilized to twice-stain grids, by touching one droplet and immediately drawing the stain off, and then placing the grid atop the second droplet to stain for 15 s. TEM images were obtained on a Tecnai TF20 transmission electron microscope (FEI) with an UltraScan 1000 (2k × 2k) CCD camera (Gatan).

### Statistical analysis of IAPP fibrils upon protein binding

The distributions of fibril diameters for IAPP amyloids, amyloid-aLac and amyloid-Lys were determined through randomly sampling 100 points on the fibrils in TEM. Measurements were undertaken using Digital Micrograph software (Gatan), and Gaussian modelling of fibril distributions was applied in Prism (GraphPad). Unpaired t-tests, utilising the Holm-Sidak method for multiple comparisons, were used to calculate statistical significance. Values wherein p < 0.05 were considered to be statistically significant.

The mesoscopic parameters of fibril persistence length (*λ*) and contour length (*l*) in the presence and absence of the proteins were analyzed with software FiberApp^21^. The FiberApp open-source code was developed from statistical polymer physics for the structural analysis of filamentous and macromolecular objects. The persistence length *λ* reflects the rigidity of a polymer and is mathematically defined via the bond correlation function (BCF) in 3D or 2D as the length over which angular correlations in the tangential direction decrease by a factor of *e*. Here the *λ* values of IAPP fibrils were estimated from the average values determined by the BCF, mean-squared end-to-end distance (MSED) and mean-squared midpoint displacement (MSMD) methods. The contour length corresponds to the end-to-end length of a polymer along its contour. The values of persistence length and contour length were obtained based on statistical analysis of 290 fibrils.

### Viability assay

IAPP, IAPP amyloids, aLac and Lys (100 μM) were incubated in Milli-Q water for 24 h at 25 °C prior to addition to cells. HUVECs (Lonza) were seeded into a black/clear bottom 96 well plate (Corning) at a density of 1.0 × 10^5^ cells/well in 200 μL EGM (Lonza) and incubated overnight (37 °C, 95% humidity, 5% CO_2_). Media was refreshed and pre-incubated IAPP, IAPP amyloids, aLac and Lys were added to final concentrations of 25 μM. For control cells, an equal volume of Milli-Q water was added to each well. Samples were added to wells in triplicate and incubated for 24 h (37 °C, 95% humidity, 5% CO_2_). Bright-field images were taken prior to calcein-AM viability testing with a Nikon TS100 bright-field microscope, equipped with a DS-Fi1 CCD camera (Nikon) and Digital Sight software (Nikon).

The calcein-AM live cell assay was used to provide quantitative data on cell viability. The calcein-AM dye is colorless in aqueous solution, but fluoresces brightly in the green spectrum when cellular esterases cleave off the AM group. In brief: media was aspirated from wells, cells were gently washed 3 × in warm HBSS (Gibco), and 100 μL aliquots of 2 μM calcein-AM dye in HBSS were added to each well. The dye was incubated with cells for 30 min at 37 °C before endpoint fluorescence was read (excitation: 485 nm; emission: 538 nm) on a Flexstation 3 plate reader (Molecular Devices). Though lysozyme is capable of non-specific esterase activity^31^, a separate control confirmed the fluorescence of calcein-AM was not affected.

### Statistical analysis of viability assay

Percentage viability of cells was calculated through direct comparison of calcein-AM fluorescence intensity with control cells (100% viable) after correcting for background fluorescence. Error bars represent the standard error of mean. A one-way ANOVA utilising Tukey’s multiple comparisons test was performed to test for statistical significance. Values were considered statistically significant if p < 0.05.

### DMD simulations

DMD is a special type of molecular dynamics algorithm where conventional continuous potentials are replaced by optimized step-wise potential functions. A more comprehensive description of the DMD algorithm was published elsewhere^32, 33^. In brief, the united-atom model represented all molecules where all heavy atoms and polar hydrogen atoms were explicitly modelled. We applied an implicit solvent model in our system. The interatomic interactions included *van der Waals*, solvation, electrostatic interactions and hydrogen bond^34^. The solvation energy was adopted by the Lazaridis-Karplus implicit solvent model, EEF1^35^. The distance- and angular-dependent hydrogen bond interaction was modelled using a reaction-like algorithm^36^. Screened electrostatic interactions were computed by the Debye-Hückel approximation. A Debye length of 1 nm was used by assuming a water dielectric constant of 80 and a monovalent electrolyte concentration of 0.1 M. The Anderson’s thermostat was used to maintain constant temperature.

In DMD simulations, the unit of time [T] is determined by units of mass [M], length [L], and energy [E], which are Dalton (1.66 × 10^−24^ g), angstrom (10^−10^ m), and kcal/mol (6.9 × 10^−22^ joule), respectively. Using the relationship $[{\rm{T}}]=[{\rm{L}}]\sqrt{[{\rm{M}}]/[{\rm{E}}]}$ as in classical MD, the time unit is approximately 50 fs^37^. All simulations were done with customized DMD software, while a similar version with the same force field is freely available via *Molecules In Action*, LLC (www.moleculesinaction.com). Our software is available upon request.

The structural coordinates for IAPP peptides were obtained from the protein data bank (Human islet amyloid peptide code 2L86, native human lysozyme code 1REX and bovine alpha lactabumin 1F6S respectively). For all these proteins and peptide as well as amyloid fibrils, basic and acidic amino acids were assigned charges corresponding to their titration states at physiological condition, i.e. Arg and Lys residues were assigned +1, Asp and Glu were assigned −1, while His was neutral. Counter ions (Cl^−^) or sodium (Na^+^) were implemented to maintain the net charge of the systems zero and accounted for possible counter–ion condensation.

We used the solid-state NMR derived constraints and the corresponding pentamer fibril models from Luca *et al*.^29^. to reconstruct a larger fibril model with two decamer protofibrils forming a two-layered fibril. Specifically, side chains of Gln10, Leu12, Asn14 and Leu16 were located inward to the beta-sheet formed by residues 28–37. The side chains of Arg11, Ala13 and Phe15 in the protofibril were buried to form the fibril. We used a central peptide from the pentamer model shared by the Tycko group and applied translational and two-fold rotational symmetries to reconstruct the larger fibril. Using the same proximity constraints, we performed DMD simulations to relax the model structure until the system’s potential energies reached equilibrium at 300 K. As the model fibril comprised of 20 IAPP monomers, we fixed most of the residues/atoms of IAPPs to reduce simulation cost. All atoms except residues 1–4 and side chains of residues 9, 11, 15, 17, 18, 20, 21 and 37 were fixed in simulations.

All simulations were conducted at 300 K. To maintain the same peptide concentration in the systems of protein-peptide ratios 6:6 and 2:2, cubic simulation box with dimensions of 137.0 Å and 95.0 Å were used correspondingly. We used a box size of 74.3 Å for the system of one protein and one peptide, 240.0 Å for 6 peptides with 48 proteins, and 150 Å for protein binding with fibril. The periodic boundary condition was applied in all simulations. For data analysis, we used an inter-atomic distance cutoff of 5.0 Å to define an atomic contact.

We used a hierarchical clustering program, oc (www.compbio.dundee.ac.uk/downloads/oc), to group similar protein binding poses with the fibril. Based on an input pair-wise distance matrix that was the center-of-mass distances of the protein on the fibril surface in our study, a hierarchical clustering algorithm iteratively joined the two closest clusters into one cluster according to the distances between two clusters. The “cluster distance” was computed based on all pairwise distances between elements of the two corresponding clusters, which can be the minimum, maximum, or the mean of all these values. In this study, we used the mean to compute the distance between two clusters. The centroid structure of each cluster was selected as the one with the smallest average distance to other elements in the cluster.

## Electronic supplementary material

Supplementary Information
