# Supplementary material for: Effects of Protein Corona on IAPP Amyloid Aggregation, Fibril Remodelling, and Cytotoxicity
Source: Sci Rep. 2017 May 26;7:2455. doi: 10.1038/s41598-017-02597-0 (PMC5446405; doi:10.1038/s41598-017-02597-0)
Supplement: Supplementary file 1 — Supplementary Information [file 41598_2017_2597_MOESM1_ESM.pdf]

Supplementary Materials

Effects of Protein Corona on IAPP Amyloid Aggregation, Fibril Remodelling, and Cytotoxicity

Emily H Pilkington,[1,#] Yanting Xing,[2,#] Bo Wang,[2] Aleksandr Kakinen,[1] Miaoyi Wang,[1] Thomas P. Davis,[1,3] Feng Ding[2*] and Pu Chun Ke[1*]*

[1]ARC Centre of Excellence in Convergent Bio-Nano Science and Technology, Monash Institute of Pharmaceutical Sciences, Monash University, 381 Royal Parade, Parkville, VIC 3052, Australia
[2]Department of Physics and Astronomy, Clemson University, Clemson, SC 29634, USA
[3]Department of Chemistry, Warwick University, Gibbet Hill, Coventry, CV4 7AL, United Kingdom

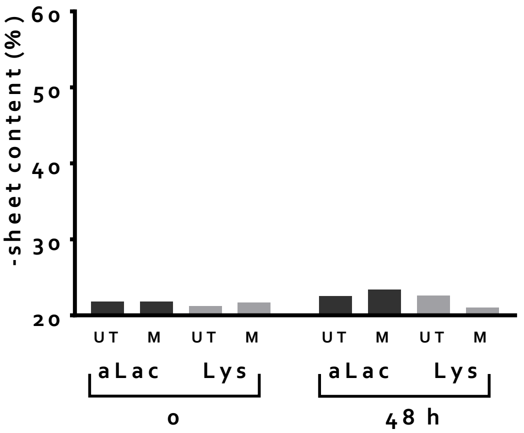

Figure S1. Circular dichroism analysis of aLac and Lys controls after 48 h. The percentage of β-sheet content within the secondary structures of aLac and Lys alone (UT) did not show any significant change when incubated at a 1:1 molar ratio with IAPP (M).

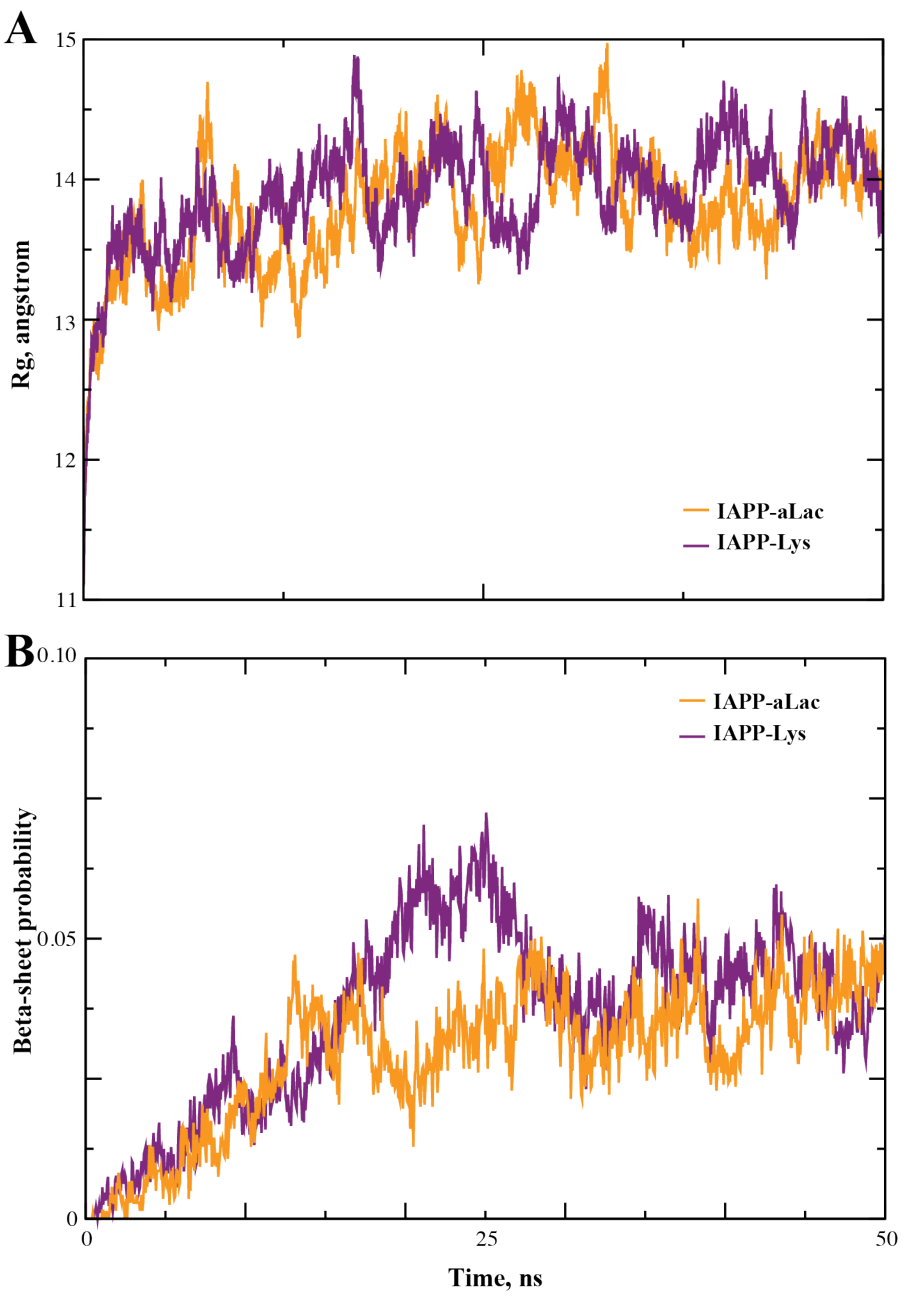

Figure S2. Binding of an IAPP monomer with aLac and Lys. (A) Radius of gyration and (B) β-sheet contents of IAPP averaged over intendant simulations were plotted as a function of time.

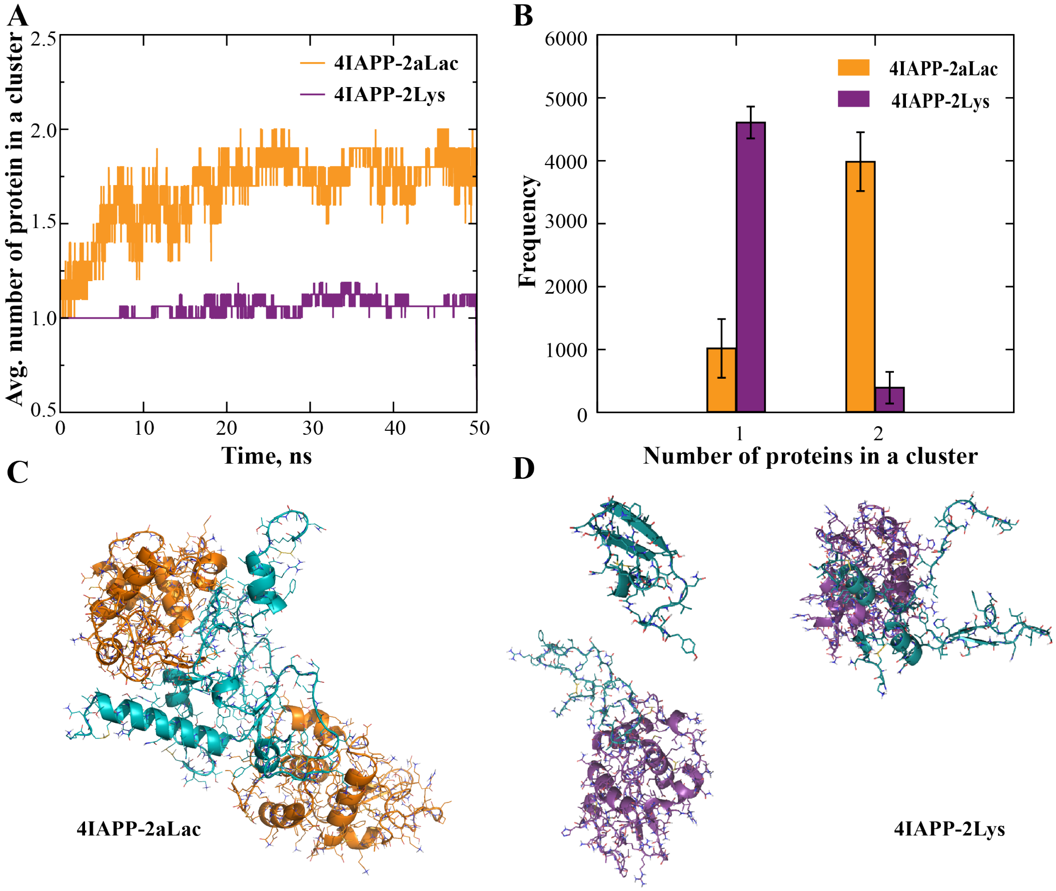

Figure S3. Binding of four IAPPs with two free proteins. (A) Average number of proteins belonging to a protein-containing cluster. (B) Histogram of the number of proteins in a protein-containing cluster. (C, D) Snapshot structures of IAPP (cyan) peptides binding with aLac (orange; C) and Lys (purple; D).

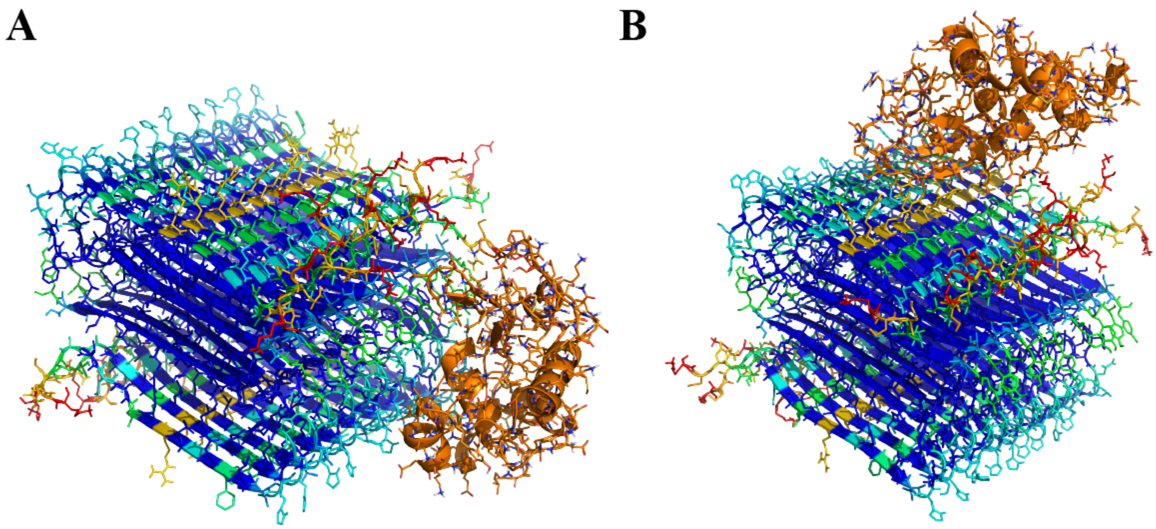

Figure S4. Centroid structures of the top two aLac-fibril binding clusters (A, B). Both aLac (orange) and peptides were shown in cartoon representation with sticks. The IAPP residues in the fibril were colored according their binding frequencies with aLac.

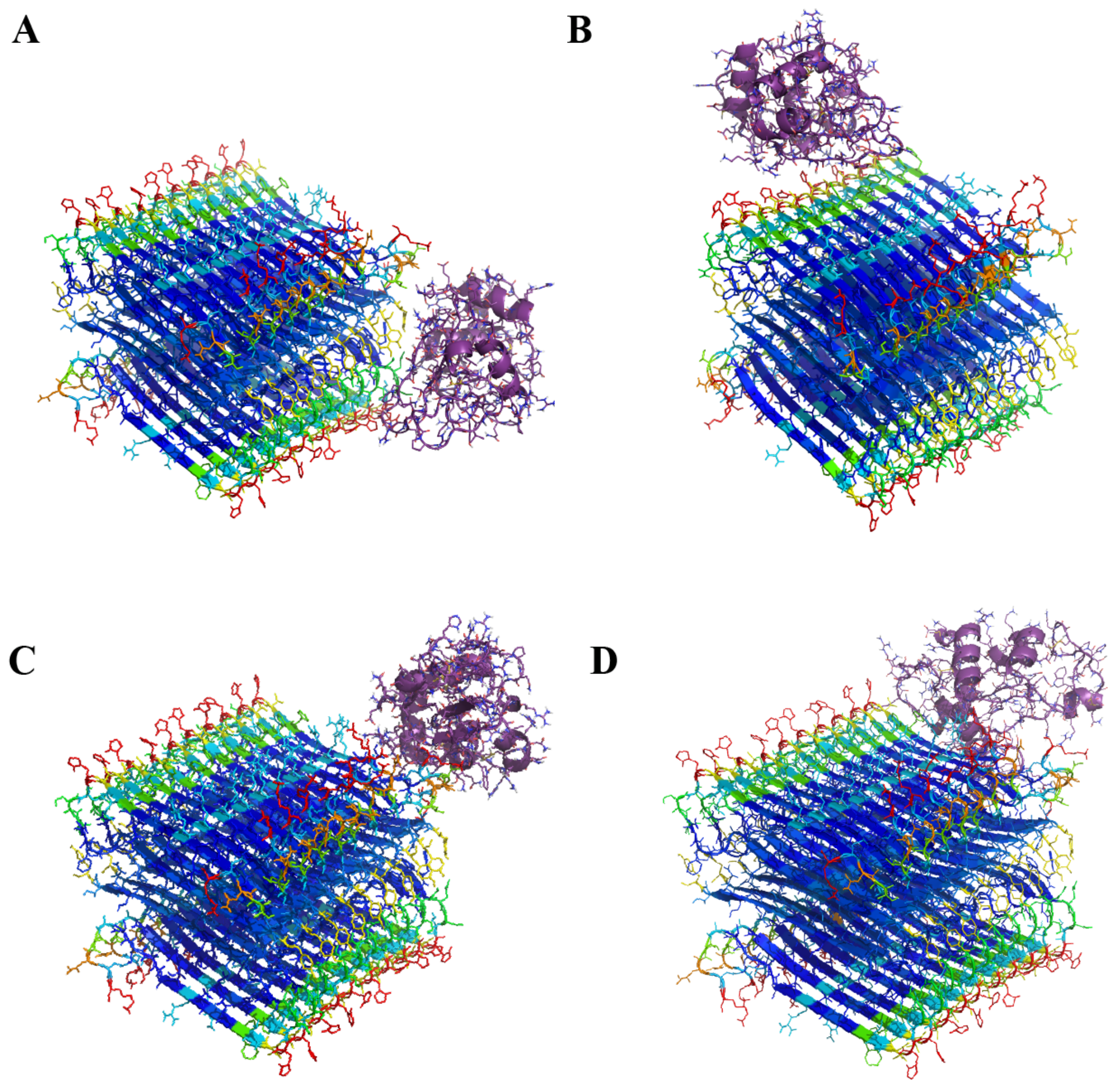

Figure S5. Centroid structures of the top four Lys-fibril binding clusters (A-D). Both Lys (purple) and peptides were shown in cartoon representation with sticks. The IAPP residues in the fibril were colored according their binding frequencies with Lys.

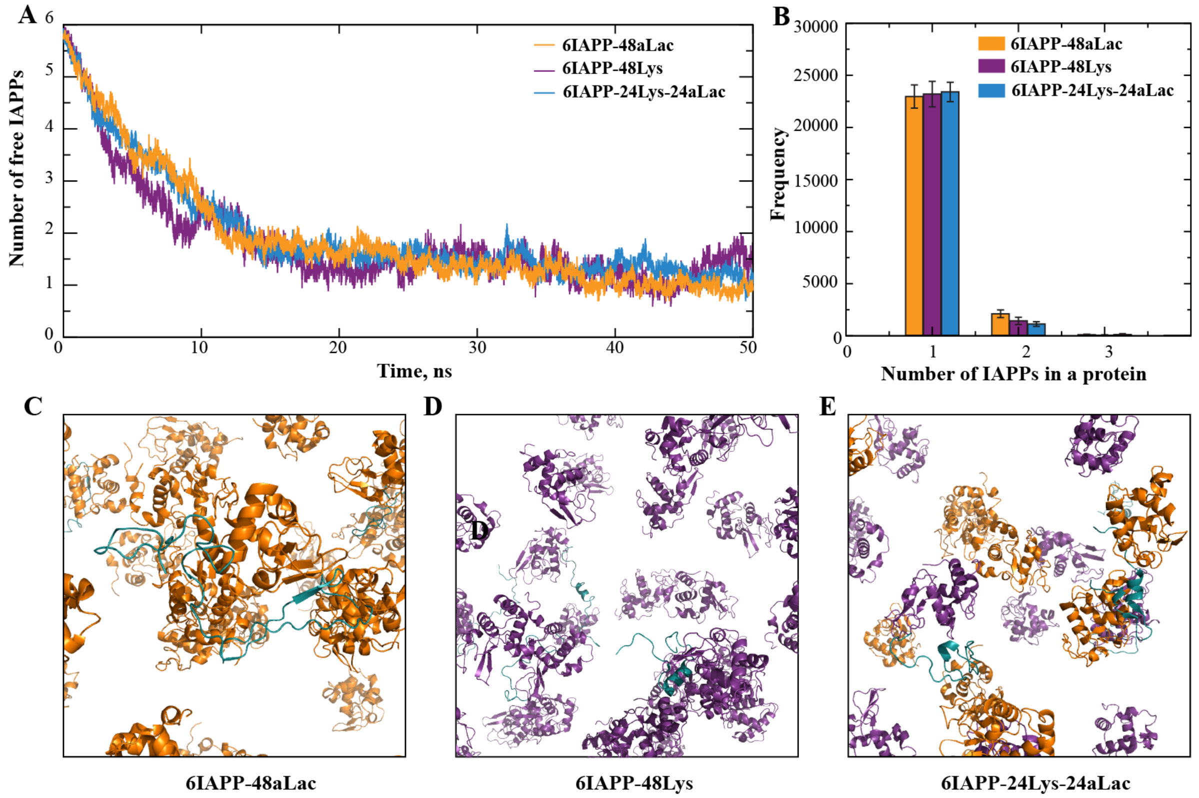

Figure S6. Binding of 6 IAPPs with 48 proteins of fixed positions. (A) Number of unbound IAPP peptides averaged over independent simulations as a function of simulation time. (B) Histogram of the number of peptides bound to either aLac, Lys, or their mixture was computed from the last 12.5 ns of corresponding simulations. (C-E) Snapshot structures of IAPP (cyan) peptides binding with either aLac (orange; C), Lys (purple; D), or their mixture (E) where both peptides and proteins were in cartoon representation.
